# Molecular basis of SARS-CoV-2 proofreading enzyme–mediated resistance to remdesivir

**DOI:** 10.1073/pnas.2519755122

**Published:** 2025-10-01

**Authors:** Yang Yang, Yu Li, Scott T. Becker, Ayesha Khan, Gloria Luo, Bin Liu, Chang Liu

**Affiliations:** ^a^Roy J. Carver Department of Biochemistry, Biophysics and Molecular Biology, Iowa State University, Ames, IA 50011; ^b^Department of Biophysics and Biophysical Chemistry, The Johns Hopkins University School of Medicine, Baltimore, MD 21205; ^c^Section of Transcription and Gene Regulation, The Hormel Institute, University of Minnesota, Austin, MN 55912

**Keywords:** remdesivir, cryo-EM, coronavirus, proofreading, drug resistance

## Abstract

SARS-CoV-2’s remarkable resistance to nucleotide analog antivirals such as remdesivir, which thwarts RNA synthesis by inhibiting viral polymerase (RdRp), challenges available therapies. We reveal that remdesivir incorporation destabilizes RdRp–RNA complex while enhancing RNA binding to the proofreading exoribonuclease (ExoN), facilitating remdesivir excision. Conserved ExoN determinants for remdesivir recognition and excision underpin ExoN-mediated resistance across all coronaviruses. These findings inform the design of next-generation antivirals and combination therapies capable of overcoming ExoN-mediated resistance.

Remdesivir is a broad-spectrum nucleotide analog (NA)-based drug that has emerged as a frontline antiviral during the COVID-19 pandemic. It inhibits SARS-CoV-2 replication by targeting the viral RNA-dependent RNA polymerase (RdRp) (1–3). Once metabolized into the active remdesivir 5′-triphosphate (RTP), it competes with the natural nucleotide adenosine 5′-triphosphate (ATP) for incorporation into viral RNA (4), causing delayed chain termination and halting RNA synthesis (1–3, 5). The characteristic 1′-cyano group (Fig. 1*A*) is the structural determinant of RTP’s RdRp stalling activity (2, 3, 5). In vitro studies and clinical trials demonstrated its efficacy in reducing viral loads (6) and improving patient outcomes (7). However, the efficacy of remdesivir is limited by the unique proofreading 3′-to-5′ exoribonuclease of coronaviruses (8), harbored in the nsp10-nsp14 complex (9, 10) (hereafter referred to as ExoN). In addition, inactivating ExoN increases viral susceptibility to remdesivir, highlighting its role as a key resistance factor (8). However, it is unclear how the remdesivir-incorporated RNA is transferred from RdRp to ExoN, both of which bind to the same 3′ end of the dsRNA substrate. Moreover, the molecular details of how ExoN recognizes remdesivir are completely lacking.

**Fig. 1:**
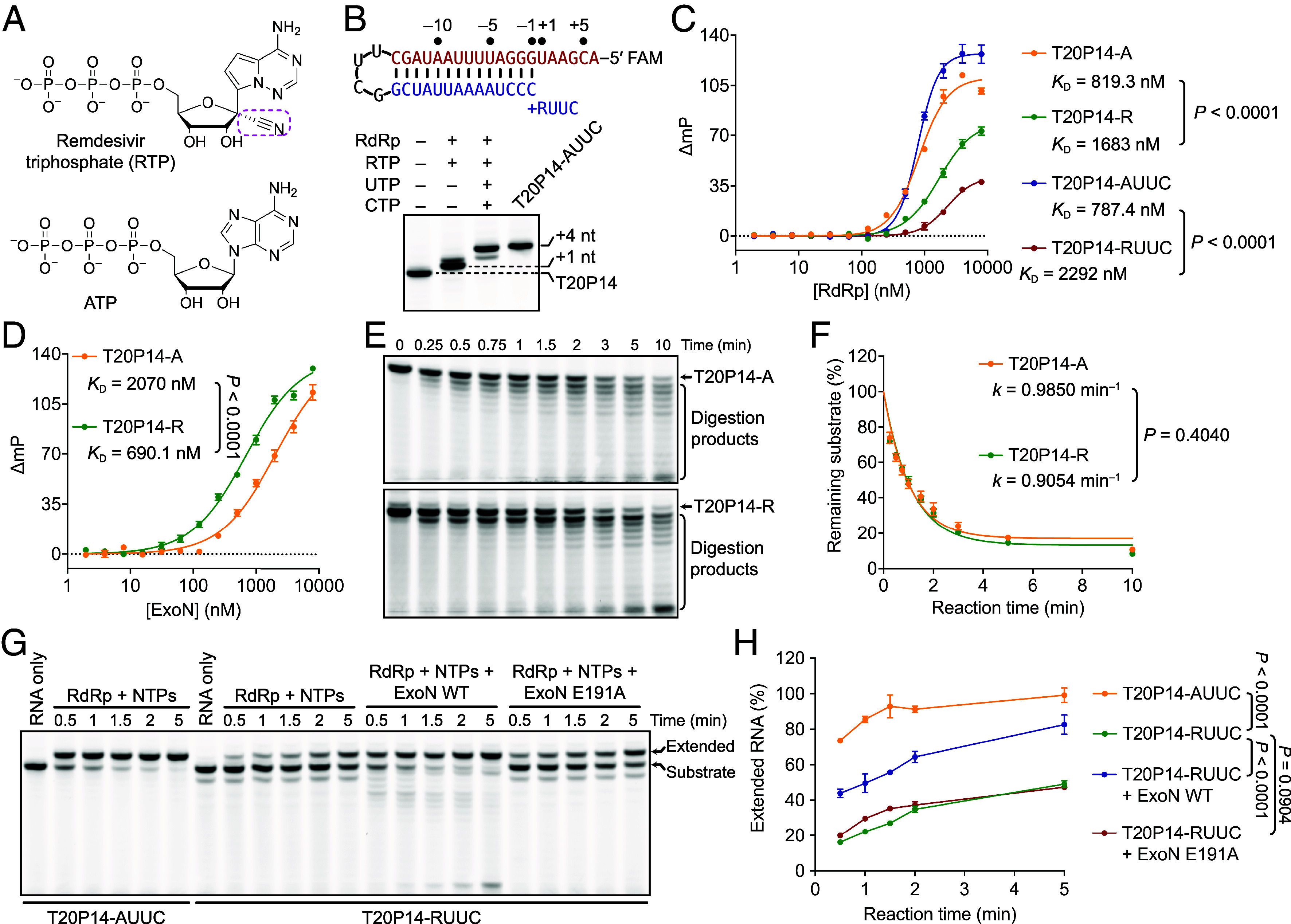
Incorporation and excision of remdesivir. (*A*) Chemical structures of RTP and ATP, with the characteristic 1′-cyano group of remdesivir highlighted (dashed magenta box). (*B*) Preparation of T20P14-R and T20P14-RUUC RNAs. T-RNA, red; P-RNA, blue. (*C*) Fluorescence polarization analysis of the binding between RdRp and T20P14-R or T20P14-RUUC versus T20P14-A or T20P14-AUUC. (*D*) Fluorescence polarization analysis of the binding between ExoN and T20P14-A or T20P14-R. Each data point in (*C* and *D*) represents the mean of nine biological replicates ± SEM. (*E*) Digestion of T20P14-A or T20P14-R by ExoN. A representative result from three biological replicates is shown. (*F*) Percentages of substrate remaining shown in (*E*) were quantified from three independent experiments and are shown as mean ± SEM. (*G*) ExoN rescues remdesivir-inhibited RNA synthesis. RNA extension reactions were performed with or without ExoN WT or E191A. (*H*) Fully extended RNA products shown in (*G*) were quantified and normalized against the control RNAs from three independent experiments and are shown as mean ± SEM.

## Results

To investigate how remdesivir affects RNA binding by RdRp and its recognition and excision by ExoN, we prepared a remdesivir 5′-monophosphate (RMP)-terminated dsRNA by incubating a hairpin RNA that comprises a 20-nucleotide (nt) template strand (T-RNA) and a 14-nt product strand (P-RNA) (designated T20P14) with purified SARS-CoV-2 RdRp and RTP (the resulting RNA is designated T20P14-R) (Fig. 1*B*). In addition, since RMP stalls RNA synthesis after RdRp incorporates three additional nucleotides (1–3, 5), we extended T20P14-R RNA by 3-nt to generate T20P14-RUUC (Fig. 1*B*). RdRp–RNA binding assay revealed that T20P14-R and T20P14-RUUC exhibited significantly lower binding affinity to RdRp than RNAs containing an adenosine at equivalent positions of remdesivir (designated T20P14-A and T20P14-AUUC, respectively) did (Fig. 1*C*), indicating that remdesivir incorporation destabilizes the RdRp–RNA interaction and may promote RdRp dissociation.

In contrast to that observed for RdRp (Fig. 1*C*), remdesivir-incorporated RNA bound significantly stronger to ExoN than adenosine-ended RNA did (Fig. 1*D*). Exoribonuclease assay revealed that ExoN digested T20P14-R as efficiently as it degraded T20P14-A (Fig. 1 *E* and *F*), demonstrating that remdesivir-terminated RNAs do not exhibit resistance to ExoN excision. While T20P14-AUUC was readily extended by RdRp, extension of T20P14-RUUC was markedly impaired (Fig. 1 *G* and *H*). However, the stalled RNA extension was substantially restored by wild-type (WT) ExoN but not a catalytically inactive mutant E191A (Fig. 1 *G* and *H*), indicating that ExoN can remove remdesivir internal of a dsRNA and additional incorporated nucleotides through multiple excision events.

**Fig. 2:**
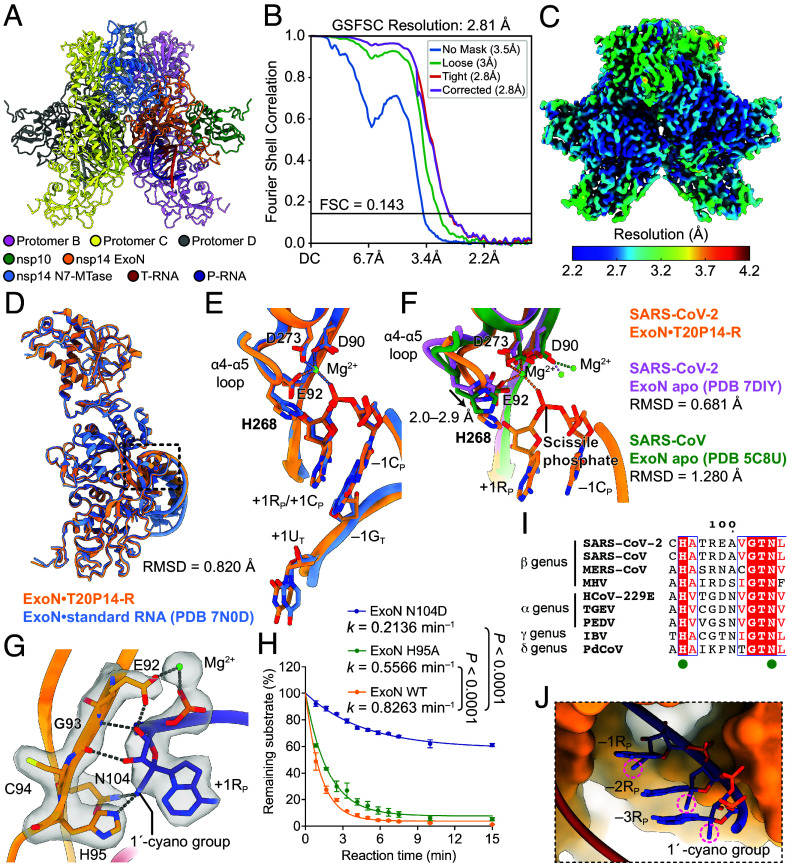
Structural basis of recognition and excision of remdesivir by ExoN. (*A*) Atomic model, (*B*) FSC curve, and (*C*) local resolution illustration of the ExoN•T20P14-R cryo-EM structure. (*D*) Overall alignment of ExoN•T20P14-R and ExoN•standard RNA complexes. (*E*) Superimposition of ExoN active site in the presence of T20P14-R or a standard RNA. (*F*) Superimposition of ExoN active sites in ExoN•T20P14-R and apo SARS-CoV-2 or SARS-CoV ExoN. (*G*) Detailed interactions between ExoN and 3′-end RMP. (*H*) Nsp14 H95A and N104D mutations greatly impair RMP excision. Each data point represents the mean of three biological replicates ± SEM. (*I*) Sequence alignment of nsp14 from different coronaviruses. H95 and N104 are indicated as green dots. (*J*) Modeling shows that the 1′-cyano groups (highlighted in magenta circles) of RMPs at –1 to –3 positions of P-RNA do not clash with ExoN.

To reveal the molecular details of remdesivir recognition and excision by ExoN, we determined the cryo-EM structure of SARS-CoV-2 ExoN E191A in complex with the T20P14-R RNA. The cryo-EM map was refined to an overall resolution of 2.8 Å, with a local resolution of the ExoN active site and remdesivir-binding pocket approaching 2.2 Å (Fig. 2 *A*–*C*).

Both the overall architecture and ExoN active site structure of the ExoN•T20P14-R are highly superimposable to those of the ExoN•standard RNA complex (11) (Fig. 2 *D* and *E*). Compared with the ExoN apo structure (12, 13), the active site residue nsp14 H268 shifts 2.0 to 2.9 Å toward the scissile phosphate (Fig. 2*F*) and adopts a catalytically competent conformation as that observed in the ExoN•standard RNA complex (11). Similar to that in the ExoN•standard RNA complex, ExoN separates the +1U_T_:+1R_P_ base-pair and flips the +1U_T_ out of RNA duplex (Fig. 2*E*). The binding mode of RMP in our cryo-EM structure differs from previous modeling based on the crystal structure of Lassa virus ExoN (14), whose active site recognizes a fully base-paired dsRNA, whereas the SARS-CoV-2 ExoN active site specifically accommodates a dsRNA with a 1-nt 3′ overhang (11) (Fig. 2*E*).

Proper positioning of the 3′-end remdesivir in the ExoN active site is mediated by many interactions with key ExoN residues. In particular, the 2′- and 3′-OH groups of the 3′-end remdesivir form multiple hydrogen bonds with nsp14 E92 and G93 (Fig. 2*G*), a hallmark of coronavirus ExoN substrate recognition also observed in ExoN•standard RNA structure (11). Besides these similarities, ExoN’s recognition of remdesivir exhibits striking differences compared to its interactions with standard nucleotides. In the ExoN•T20P14-R complex, the hydrogen bond between nsp14 H95 and the 3′-end nucleobase observed in the ExoN•standard RNA structure (11) is lost, potentially due to steric restraints posed by the 1′-cyano group of remdesivir (Fig. 2*G*). Moreover, the 1′-cyano group of remdesivir projects between nsp14 H95 and N104, forming two hydrogen bonds with these residues (Fig. 2*G*). These additional interactions provided by the 1′-cyano group of remdesivir likely compensate for the loss of the nucleobase-mediated hydrogen bond and contribute to the higher ExoN-binding affinity of T20P14-R than that of T20P14-A (Fig. 1*D*). Our mutagenesis analysis showed that either H95A or N104D mutation of nsp14, which disrupts their hydrogen-bonding interactions with the 1′-cyano group of remdesivir, significantly weakens the excision of the 3′-end remdesivir from RNA (Fig. 2*H*), supporting the important roles of these two residues in remdesivir recognition and excision. Notably, H95 and N104 are completely conserved across all coronavirus genera (Fig. 2*I*), suggesting a shared mechanism for the recognition and excision of remdesivir by ExoNs among different coronaviruses.

In addition, structural modeling suggests that RMPs at –1 to –3 positions of the P-RNA strand are also well accommodated in the ExoN•RNA complex (Fig. 2*J*), explaining the efficient removal of RMP internal of a dsRNA as revealed by our (Fig. 1*G*) and previous studies (15). Taken together, our study uncovers the molecular basis for ExoN-mediated recognition of incorporated remdesivir and ExoN-conferred coronavirus resistance to remdesivir.

## Discussion

Our work not only reveals efficient excision of both 3′ end and internal remdesivir by SARS-CoV-2 ExoN, as previously reported (15), but also elucidates the dynamic interplay among remdesivir-incorporated RNA, RdRp, and ExoN. We show that incorporation of remdesivir into RNA destabilizes its engagement with RdRp while enhancing its binding to ExoN. Such reciprocal changes in RNA-binding dynamics likely facilitate the transfer of remdesivir-incorporated RNA to ExoN for subsequent excision, advancing our understanding of coronavirus resistance to remdesivir. Additionally, while previous model suggested that incorporated remdesivir remains stuck in RdRp (14) and thus inaccessible to ExoN, our findings indicate that ExoN readily removes internal remdesivir and rescues stalled RNA synthesis in the presence of RdRp. Furthermore, our structural insights into remdesivir recognition by ExoN will guide the design of next-generation NA-based antivirals capable of overcoming ExoN-mediated resistance, ultimately improving the antiviral arsenal against SARS-CoV-2 and other coronaviruses.

## Materials and Methods

All SARS-CoV-2 proteins were expressed in *Escherichia coli* BL21 Star (DE3) and purified through HisTrap, Heparin, and size-exclusion chromatography column, consecutively. T20P14-R RNA was produced by in vitro transcription. Cryo-EM data were collected on a Titan Krios electron microscope and processed using cryoSPARC. See *SI Appendix*, *Extended Methods* for details.

## Supplementary Material

Appendix 01 (PDF)

## Data Availability

The coordinate and cryo-EM map of the ExoN•T20P14-R have been deposited with accession codes PDB 9Q1J (16) and EMD-72127 (17), respectively.
