# Supplementary material for: Molecular basis of SARS-CoV-2 proofreading enzyme–mediated resistance to remdesivir
Source: Proc Natl Acad Sci U S A. 2025 Oct 1;122(40):e2519755122. doi: 10.1073/pnas.2519755122 (PMC12519199; doi:10.1073/pnas.2519755122)
Supplement: Supplementary file 1 — Appendix 01 (PDF) [file pnas.2519755122.sapp.pdf]

PNAS

Supporting Information for

Molecular basis of SARS-CoV-2 proofreading enzyme-mediated resistance to remdesivir

Yang Yang[1],*, Yu Li[1], Scott T. Becker[1], Ayesha Khan[1], Gloria Luo[2], Bin Liu[3],*, Chang Liu[2],*

* Corresponding authors: Yang Yang, Bin Liu, Chang Liu
Email: yan9yang@iastate.edu (Y.Y.); liu00794@umn.edu (B.L.); cliu207@jhmi.edu (C.L.)

This PDF file includes:

Extended Methods
SI References

Extended Methods

Protein expression and purification

The full-length genes of SARS-CoV-2 nsp7, nsp8, and nsp12 (GenBank accession number NC_045512.2) were chemically synthesized with codon optimization for expression in *Escherichia coli* (*E. coli*) (GENEWIZ). The –1 ribosomal frameshifting that naturally occurs in the virus to produce nsp12 was corrected in the synthesized gene. The genes were fused to an N-terminal His_6-Smt3 tag and were cloned into a pET21a vector (Millipore Sigma) between NdeI and XhoI restrictive sites. The genes of SARS-CoV-2 nsp10 were chemically synthesized with codon optimization for expression in *Escherichia coli* (*E. coli*) (Integrated DNA Technologies). The gene of SARS-CoV-2 nsp14 was requested from Addgene. The SARS-CoV-2 nsp14 genes were cloned into a pETDuet-1 vector with an N-terminal His_6-Smt3 tag between the NcoI and HindIII restrictive sites. The SARS-CoV-2 nsp10 genes were cloned into a pETDuet-1 vector between the NdeI and XhoI restrictive sites. E191A mutation of nsp14 was introduced by site-directed mutagenesis. The mutation was confirmed by Sanger sequencing.

All proteins were overexpressed in *E. coli* BL21 Star (DE3) (ThermoFisher Scientific) at 17 °C for 18 h. Cells were resuspended in buffer A (50 mM 4-(2-hydroxyethyl)-1-piperazine ethanesulfonic acid (HEPES), pH 7.5, 200 mM NaCl, 5% glycerol, 1 mM β-mercaptoethanol (β-ME), 20 mM imidazole) and lysed using a sonicator (QSonica). The cell lysate was cleared by centrifugation at 19,500 rpm using a JA-25.50 rotor (Beckman Coulter) for 1 h at 4 °C. The clarified cell lysate was loaded onto a HisTrap HP affinity chromatography column (Cytiva Life Sciences) and eluted through a linear gradient from 100% buffer A to 40% buffer A mixed with 60% buffer B (50 mM HEPES, pH 7.5, 500 mM NaCl, 5% glycerol, 1 mM β-ME, 500 mM imidazole). Eluted protein samples were loaded onto a HiTrap Heparin HP column (Cytiva Life Sciences) and eluted with buffer C (20 mM HEPES, pH 7.0, 1 M NaCl, 2 mM β-ME), followed by overnight incubation with Ulp1 protease to remove the N-terminal His_6-Smt3 tag. All proteins were subsequently purified by size-exclusion chromatography (SEC) on a HiLoad 16/600 Superdex 200 pg column (Cytiva Life Sciences) in buffer D (20 mM HEPES, pH 7.5, 300 mM NaCl, 4 mM $MgCl_2$, 1 mM Tris(2-carboxyethyl) phosphine hydrochloride (TCEP-HCl)). ExoN complex was assembled by mixing nsp10 and nsp14 in a 1:1 molar ratio at room temperature for 30 min. RdRp complex was assembled by mixing nsp12, nsp7, and nsp8 in a 1:1:2 molar ratio at room temperature for 30 min.

In vitro transcription and RNA purification

The DNA template for *in vitro* transcription of the T20P14 RNA was generated by annealing two DNA oligonucleotides with complementary sequences (5'- CAG <u>TAA TAC GAC TCA CTA TAG</u> GGA ATG GGA TTT TAA TAG CTT CGG CTA TTA AAA TCC C -3' and 5'-[G_m][G_m]G ATT TTA ATA GCC GAA GCT ATT AAA ATC CCA TTC CCT ATA GTG AGT CGT ATT ACT G-3') in a 1:1 molar ratio. T7 RNA polymerase (RNAP) ϕ6.5 promoter sequence in the non-template

DNA strands is underlined. Two nucleotides denoted [G_m] on the 5' end of the template DNA strand are 2'-*O*-methylated to improve the 3'-end homogeneity of the RNA transcripts (1).

In vitro transcription reaction (5 mL) was assembled using 2 nmol annealed DNA template, 500 μg T7 RNAP and 200 μl 25× RNAsecure RNase inactivation reagent (ThermoFisher Scientific) in 1× reaction buffer (80 mM HEPES, pH 7.5, 24 mM $MgCl_2$, 40 mM DTT, 2 mM spermidine, 4 mM of each NTP) and incubated at 37 °C for 2 h. The reaction mixture was centrifugation at 4,000 × g for 10 min at 4 °C to remove pyrophosphate precipitate and subsequently quenched by adding ethylenediaminetetraacetic acid (EDTA) to a final concentration of 50 mM. The RNA transcripts were extracted with phenol:chloroform:isoamyl alcohol (25:24:1) (ThermoFisher Scientific) three times, followed by purification on a Sephadex G-25 PD-10 desalting column (Cytiva Life Sciences) and a HiLoad 16/600 Superdex 200 pg SEC column in buffer E (10 mM HEPES, pH 7.0, 50 mM NaCl).

5'-FAM-labeled RNAs are chemically synthesized (MilliporeSigma), followed by purification on a Superdex 200 Increase 10/300 GL SEC column (Cytiva Life Sciences) in buffer E.

To generate nucleotide analog-incorporated RNAs, purified T20P14 RNAs were incubated with pre-assembled SARS-CoV-2 RdRp and 0.3 mM of remdesivir triphosphate (MedChemExpress) at 37 °C for 2 h in buffer F (25 mM HEPES, pH 7.5, 75 mM NaCl, 4 mM $MgCl_2$, and 1 mM TCEP). The reaction mixture was centrifugation at 4,000 × g for 10 min at 4 °C to remove pyrophosphate precipitate and subsequently quenched by adding EDTA to a final concentration of 50 mM. The RNA transcripts were extracted with phenol:chloroform:isoamyl alcohol (25:24:1) three times to completely remove the RdRp, followed by purification on a Superdex 200 Increase 10/300 GL SEC column in buffer E. To generate T20P14-RUUC RNA, purified T20P14-R RNA was incubated with pre-assembled SARS-CoV-2 RdRp, 0.3 mM UTP, and 0.3 mM CTP at 37 °C for 2 h in buffer F. The reaction mixture was centrifugation at 4,000 × g for 10 min at 4 °C and subsequently quenched by adding EDTA to a final concentration of 50 mM. The RNA transcripts were extracted with phenol:chloroform:isoamyl alcohol (25:24:1) three times, followed by purification on a Superdex 200 Increase 10/300 GL SEC column in buffer E.

Fluorescence polarization assays

FAM-labeled T20P14 series of RNAs at a final concentration of 6 nM were incubated with a 2x serial dilution series (ranging from 1.953 nM to 8 μM) of pre-assembled SARS-CoV-2 RdRp WT or ExoN E191A mutant complex at room temperature in buffer G (25 mM HEPES, pH 7.5, 50 mM NaCl, 4 mM $MgCl_2$, and 1 mM TCEP) in a 384-well plate. Fluorescence polarization was measured on a Victor Nivo multimode microplate reader (Revvity) with the excitation and emission wavelengths of 480 nm and 530 nm, respectively. Changes in fluorescence polarization (ΔmP) upon protein binding were plotted against RdRp WT or ExoN E191A concentration in GraphPad Prism. The data for ExoN•RNA binding were fitted using a custom "One site-specific binding with ligand depletion" model (2) [$Y = B_{max} \times (X - F \times Y/B_{max})/(K_D + X - F \times Y/B_{max})$], where X is the total protein concentration, F is the total fluorescence probe concentration, Y is the change of fluorescence polarization from the RNA-only control group,

B_{max} is maximum binding in the same units as Y, and K_D is the dissociation constant in the same unit as X] to determine the K_D values and 95% confidence intervals. The data for RdRp•RNA binding were fitted using a custom "Specific binding with variable slope and ligand depletion" model [$Y = B_{max} \times (X - F \times Y/B_{max})^n / (K_D^n + (X - F \times Y/B_{max})^n)$, where n is the Hill slope] to determine the K_D values and 95% confidence intervals. Statistical analyses for comparing the best-fit K_D values between each group were performed using the extra sum-of-squares F test.

Exoribonucleolytic digestion of T20P14-A and T20P14-R

WT SARS-CoV-2 nsp10•nsp14 complex at a final concentration of 20 nM was incubated with FAM-T20P14-A or FAM-T20P14-R with a final concentration of 3 μM at 37 °C in buffer G. The reactions were stopped at 15 s, 30 s, 45 s, 60 s, 90 s, 120 s, 3 min, 5 min, and 10 min, respectively, by adding an equal volume of 2× TBE-Urea sample buffer supplemented with 50 mM EDTA and heating at 75 °C for 5 min. The cleavage products were resolved on denaturing 16% polyacrylamide gels and visualized by fluorescent imaging on a ChemiDoc MP imager (Bio-Rad). The RNA band corresponding to the substrate RNA at each reaction time point was quantified using Image Lab Software Suite (Bio-Rad). Percentages of substrate RNAs remaining were plotted against their respective reaction times in GraphPad Prism. The results were subjected to curve-fitting using the One-phase decay model to determine the rate constant (k) of RNA digestion for each reaction. Statistical analyses for comparing the best-fit rate constant (k) values between each group were performed using the extra sum-of-squares F test.

Rescue of stalled RNA synthesis assay

FAM-labeled T20P14 series of RNAs at a final concentration of 1 μM were incubated with pre-assembled SARS-CoV-2 RdRp at a final concentration of 1.25 μM at 30 °C for 5 min in buffer F. The reactions were started by adding NTPs, a mixture of NTPs and 10 nM SARS-CoV-2 nsp10•nsp14 WT, or a mixture of NTPs and 10 nM SARS-CoV-2 nsp10•nsp14 E191A mutant. NTPs were supplied at a final concentration of 4 μM each. The reactions were stopped at different time points by adding an equal volume of 2× TBE-Urea sample buffer supplemented with 50 mM EDTA and heating at 75 °C for 5 min. RNA products were resolved on denaturing 16% polyacrylamide gels and visualized by fluorescent imaging on a ChemiDoc MP imager (Bio-Rad). The RNA band corresponding to the substrate RNA at each reaction time point was quantified using Image Lab Software Suite (Bio-Rad).

Complex assembly of SARS-CoV-2 ExoN with RMP-incorporated RNA

The complex of SARS-CoV-2 ExoN and RMP-terminated RNA was reconstituted by mixing SARS-CoV-2 nsp10•nsp14 E191A mutant complex with T20P14-A plus P6 RNA (5'-UUCCCC-3', chemically synthesized from MilliporeSigma) in a 1:2:2 molar ratio and incubating the mixture at 30 °C for 30 min in buffer G. The assembled SARS-CoV-2 ExoN•RNA complex was purified using a Superdex 200 Increase 10/300 GL SEC column in buffer G. The

chromatography fractions corresponding to the ExoN•RNA complex were collected for subsequent single-particle cryo-EM analysis.

Cryo-EM sample preparation and data acquisition

Purified SARS-CoV-2 ExoN•RNA complexes (A260=3) were mixed with 8mM of 3-([3-Cholamidopropyl]dimethylammonio)-2-hydroxy-1-propanesulfonate (CHAPSO) immediately before grid preparation. 3.5 µl of each complex was applied to freshly glow-discharged Quantifoil 300 mesh holey carbon grids with R1.2/1.3 hole pattern (Electron Microscopy Sciences). Grids were blotted for 5 s at 22 °C under 100% relative humidity and plunge-frozen in liquid nitrogen-cooled liquid ethane. The cryo-EM dataset, which contains 10,859 dose-fractioned movies, was collected on a Titan Krios electron microscope (ThermoFisher Scientific) operated at 300 kV equipped with a BioQuantum K3 detector (Gatan, Inc.) at the Hormel Institute, University of Minnesota. The movie frames were collected at a nominal magnification of 130,000×, corresponding to 0.6563 Å per pixel, at a dose rate of 11.1 e^- per physical pixel per second, with a defocus range of –1.0 to –2.0 µm. The total exposure time for each movie is 2 s, thus resulting in a total accumulated dose of 51.54 $e^-/Å^2$, which was fractionated into 40 frames.

Cryo-EM image processing

Dose-fractioned cryo-EM movies were imported into cryoSPARC (3) for patch-based motion correction and patch-based CTF estimation, followed by blob picking and Topaz picking (4), which resulted in 895,129 and 530,375 particles, respectively. The picked particles were subjected to three rounds of 2D classifications to remove junk particles. 110,586 particles in good 2D classes of the tetrameric form of the ExoN•T20P14-R complex were selected for non-uniform refinement (5) of the ExoN•T20P14-R complex, yielding a cryo-EM map at a resolution of 3.2 Å. After that, particles with a defocus value higher than 2.5 µm were removed. The remaining 102,206 particles were then subjected to iterative rounds of CTF refinement (6), Reference-based motion correction (7, 8), and non-uniform refinement (5) with *C*2 symmetry to generate the final cryo-EM map at a resolution of 2.8 Å.

The final non-uniform refined map for the ExoN•T20P14-R complex was further improved by density modification using Phenix Resolve (9) without supplying a structural model to avoid model bias. The overall map resolution was calculated based on the Fourier shell correlation (FSC) cutoff at 0.143 between two half-maps, after applying a soft mask to exclude the bulk solvent region. The map was sharpened automatically by applying a sharpening *B* factor of 84.8 Å during non-uniform refinement and post-processed using DeepEMhancer (10). The raw map, automatically sharpened map, Resolve density-modified map, and DeepEMhancer-processed map were used as cross-references during model building. Local resolution estimation was calculated from the two half-maps in cryoSPARC and visualized in UCSF ChimeraX (11). Direction FSC analysis for the cryo-EM map by the Orientation Diagnostics tool (12, 13) in cryoSPARC yielded a conical FSC Area Ratio (cFAR)

value of 0.80, which is well above the anisotropy threshold of 0.5 and indicates the absence of preferred orientation of the cryo-EM data.

Cryo-EM model building and refinement

The cryo-EM structure of the tetrameric form of SARS-CoV-2 ExoN•RNA complex (7N0D) was docked into the ExoN•T20P14-R complex map as a rigid body and was then flexibility fitted (14) into the ExoN•RNA complex map. The protein and RNA subunits were manually rebuilt in *Coot* (15). The resolution and density features of the cryo-EM maps are of sufficiently high quality for the unambiguous assignment of protein and RNA registers in the complexes. The final structural model contains 2572 protein residues, 56 nucleotides, and 26 ligands. Nsp10 residues 132–139, nsp14 residues 1, 455–464, and 524–527, and T20P14-R RNA residues 15–30 are disordered and are not included in the final structural model. The atomic model was refined using Phenix real-space refinement (16) with secondary structure restraints, rotamer restraints, and Ramachandran restraints. The model-map resolution was determined to be 2.9 Å with an FSC threshold of 0.5. The final structure was validated with MolProbity (17). The final structural model has a MolProbity score of 1.03 and a Clashscore of 2.12. Among the 2572 protein residues, none of them have poor side chain rotamers; 97.80% of them are in the Ramachandran plot favored region, 2.20% in the allowed region, and 0.00% in the disallowed region. Molecular representations were prepared using UCSF ChimeraX. Sequence alignments were performed in Clustal Omega (18) and displayed using the online server of Espript 3.0 (19).

Mutagenesis analysis of ExoN•remdesivir interactions

WT SARS-CoV-2 nsp10•nsp14 complex or nsp10•nsp14 complex carrying nsp14 H95A or N104D mutants at a final concentration of 8 nM was incubated with FAM-T20P14-R RNA with a final concentration of 1 μM at 37 °C in buffer G. The reactions were stopped at 50 s, 100 s, 150 s, 200 s, 250 s, 300 s, 350 s, 400 s, 450 s, 600 s, and 900 s, respectively, by adding an equal volume of 2× TBE-Urea sample buffer supplemented with 50 mM EDTA and heating at 75 °C for 5 min. The cleavage products were resolved on denaturing 18% polyacrylamide gels and visualized by fluorescent imaging on a ChemiDoc MP imager (Bio-Rad). The RNA band corresponding to the substrate RNA at each reaction time point was quantified using Image Lab Software Suite (Bio-Rad). Percentages of substrate RNAs remaining were plotted against their respective reaction times in GraphPad Prism. The results were subjected to curve-fitting using the One-phase decay model to determine the rate constant (k) of RNA digestion for each reaction. Statistical analyses for comparing the best-fit rate constant (k) values between each group were performed using the extra sum-of-squares F test.

SI References

1. C. Kao, M. Zheng, S. Rudisser, A simple and efficient method to reduce nontemplated nucleotide addition at the 3 terminus of RNAs transcribed by T7 RNA polymerase. *RNA* **5**, 1268-1272 (1999).
2. M. A. Esler *et al.*, A compact stem-loop DNA aptamer targets a uracil-binding pocket in the SARS-CoV-2 nucleocapsid RNA-binding domain. *Nucleic Acids Res* **52**, 13138-13151 (2024).
3. A. Punjani, J. L. Rubinstein, D. J. Fleet, M. A. Brubaker, cryoSPARC: algorithms for rapid unsupervised cryo-EM structure determination. *Nat Methods* **14**, 290-296 (2017).
4. T. Bepler *et al.*, Positive-unlabeled convolutional neural networks for particle picking in cryo-electron micrographs. *Nat Methods* **16**, 1153-1160 (2019).
5. A. Punjani, H. Zhang, D. J. Fleet, Non-uniform refinement: adaptive regularization improves single-particle cryo-EM reconstruction. *Nat Methods* **17**, 1214-1221 (2020).
6. J. Zivanov, T. Nakane, S. H. W. Scheres, Estimation of high-order aberrations and anisotropic magnification from cryo-EM data sets in RELION-3.1. *IUCrJ* **7**, 253-267 (2020).
7. J. Zivanov, T. Nakane, S. H. W. Scheres, A Bayesian approach to beam-induced motion correction in cryo-EM single-particle analysis. *IUCrJ* **6**, 5-17 (2019).
8. T. Grant, N. Grigorieff, Measuring the optimal exposure for single particle cryo-EM using a 2.6 A reconstruction of rotavirus VP6. *Elife* **4**, e06980 (2015).
9. T. C. Terwilliger, S. J. Ludtke, R. J. Read, P. D. Adams, P. V. Afonine, Improvement of cryo-EM maps by density modification. *Nat Methods* **17**, 923-927 (2020).
10. R. Sanchez-Garcia *et al.*, DeepEMhancer: a deep learning solution for cryo-EM volume post-processing. *Commun Biol* **4**, 874 (2021).
11. T. D. Goddard *et al.*, UCSF ChimeraX: Meeting modern challenges in visualization and analysis. *Protein Sci* **27**, 14-25 (2018).
12. Y. Z. Tan *et al.*, Addressing preferred specimen orientation in single-particle cryo-EM through tilting. *Nat Methods* **14**, 793-796 (2017).
13. P. R. Baldwin, D. Lyumkis, Non-uniformity of projection distributions attenuates resolution in Cryo-EM. *Prog Biophys Mol Biol* **150**, 160-183 (2020).
14. T. I. Croll, ISOLDE: a physically realistic environment for model building into low-resolution electron-density maps. *Acta Crystallogr D Struct Biol* **74**, 519-530 (2018).
15. A. Casanal, B. Lohkamp, P. Emsley, Current developments in Coot for macromolecular model building of Electron Cryo-microscopy and Crystallographic Data. *Protein Sci* **29**, 1069-1078 (2020).
16. P. V. Afonine *et al.*, Real-space refinement in PHENIX for cryo-EM and crystallography. *Acta Crystallogr D Struct Biol* **74**, 531-544 (2018).
17. V. B. Chen *et al.*, MolProbity: all-atom structure validation for macromolecular crystallography. *Acta Crystallogr D Biol Crystallogr* **66**, 12-21 (2010).

18. F. Sievers, D. G. Higgins, Clustal omega. *Curr Protoc Bioinformatics* **48**, 3 13 11-13 13 16 (2014).

19. X. Robert, P. Gouet, Deciphering key features in protein structures with the new ENDscript server. *Nucleic Acids Res* **42**, W320-324 (2014).
